# The Lateral Occipital Complex shows no net response to object familiarity

**DOI:** 10.1167/16.11.3

**Published:** 2016-09-06

**Authors:** Eshed Margalit, Manan P. Shah, Bosco S. Tjan, Irving Biederman, Brenton Keller, Rorry Brenner

**Affiliations:** eshed.margalit@gmail.com; mshah726@gmail.com; btjan@usc.edu; bieder@usc.edu; brenton8907@gmail.com; rorry.brenner@usc.edu; Neuroscience Program, University of Southern California, Los Angeles, CA, USA; Department of Psychology, University of Southern California, Los Angeles, CA, USA

**Keywords:** *Lateral Occipital Complex*, *object representation*, *object familiarity*

## Abstract

In 1995, Malach et al. discovered an area whose fMRI BOLD response was greater when viewing intact, familiar objects than when viewing their scrambled versions (resembling texture). Since then hundreds of studies have explored this late visual region termed the Lateral Occipital Complex (LOC), which is now known to be critical for shape perception (James, Culham, Humphrey, Milner, & Goodale, 2003). Malach et al. (1995) discounted a role of familiarity by showing that “abstract” Henry Moore sculptures, unfamiliar to the subjects, also activated this region. This characterization of LOC as a region that responds to shape independently of familiarity has been accepted but never tested with control of the same low-level features. We assessed LOC's response to objects that had identical parts in two different arrangements, one familiar and the other novel. Malach was correct: There is no net effect of familiarity in LOC. However, a multivoxel correlation analysis showed that LOC does distinguish familiar from novel objects.

## Introduction

Malach et al. (1995) discovered that the lateral occipital cortex (LO) and the posterior fusiform gyrus (pFs), cortical areas that they termed the Lateral Occipital Complex (LOC), yielded greater fMRI BOLD responses when viewing intact images of familiar objects than when viewing their scrambled versions resembling texture. LOC was subsequently shown to be critical for shape-based object recognition in that an individual, DF, who had suffered bilateral lesions to LOC as a result of carbon monoxide poisoning, became severely shape agnosic but showed no deficit in her perception of color, surface texture, and material properties (James et al., 2003).

Malach et al. discounted a role of familiarity by showing that “abstract” Henry Moore sculptures, unfamiliar to his subjects, also activated LOC. Although such a comparison does indicate that intact images produce greater LOC activation than their scrambled versions, it is not clear, without control for low-level stimulus features, whether variations in familiarity produce any differential activation in that region. It could be, for example, that the individual features of the Moore sculptures produced greater activation than the features of the familiar objects selected by Malach et al., which could have balanced out greater activation of familiar over novel shapes.

**Figure 1:**
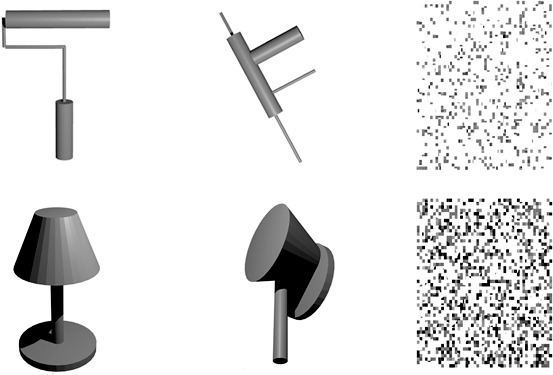
Examples of familiar, novel, and scrambled objects, with one set of stimuli in each row. Each novel object (middle column) contained the same geons as its familiar version (left column) but in different between-geon relations. The scrambled version of each image (right column) was created by permuting blocks of 3 × 3 pixels in the familiar version. All the images were rendered in gray. The paint roller in the first row is an example of an asymmetric object, whereas the lamp in the second row is a symmetric object.

Attempts have been made to identify the regions of the brain that respond to object familiarity. However, the stimuli used in such experiments either did not target shape specifically or created images that were readily interpretable as an incongruous pairing of familiar categories, e.g., a bird with a fox's head or a banana joined to a red pepper, as in Zhang, Liu, and Zhang's (2013) study in which the stimuli combined halves of familiar natural (plant or animal) colored photos. They did not compare performance with the scrambled versions of these stimuli so LOC was not localized. Zhang et al. reported that the left precuneus was more activated by the novel combinations of the objects. A PET study by Kanwisher, Woods, Iacoboni, and Mazziotta (1997) showed that line drawings of familiar objects as well as novel hand drawn intact objects with some matching of the line characteristics did result in greater activity than their scrambled versions in areas that would overlap with that of the present study, but there was no direct way to compare individual novel and intact images with respect to their part composition. In the present investigation, gray-level rendered images of novel objects were created by spatially rearranging the simple parts (geons) found in familiar objects so they were not readily identifiable as any familiar or combination of familiar object models (Figure 1). There is strong evidence that LOC represents objects in terms of the object's parts and relations (Hayworth & Biederman, 2006; Hayworth et al., 2011; Lescroart & Biederman, 2013). Hayworth and Biederman (2006) used an adaptation design with complementary pairs of contour-deleted line drawings of familiar objects in which a complementary pair could have either half the intact parts of an object (“Parts Deleted,” PD) or all the parts but with every other line and vertex deleted from each part (“Local Feature Deleted,” LFD). In both the PD and the LFD conditions, the complement had the remaining half of the contour so that superimposing members of a complementary pair would produce an image of an intact object without any overlap of contour. All the visual priming, assessed as maintenance of the adaptation of the BOLD response, could be attributed to a representation of an object's parts and none to its local image features (e.g., vertices and contour) or basic- or subordinate-level concepts. That is, no adaptation was evident between complementary pairs with different parts, and completely changing the local features so that not a single vertex or line was present across both members of a complementary LFD pair failed to produce any release from adaptation. These results were completely consistent with the results of a behavioral priming study reported by Biederman and Cooper (1991) in which participants viewed briefly presented, masked LFD images in two blocks of trials for speeded naming. On the second block, complementary pairs of images yielded the same degree of priming, measured as the reduction in naming reaction times and error rates, as the identical images shown on the first block. The naming of the identical and LFD complements were both named substantially faster and more accurately than same-name, different shaped exemplars, indicating that the priming was visual rather than lexical or conceptual. PD complements, on the other hand, produced no visual priming, in that identification performance of such complements was equivalent to same name, different shaped exemplars, both of which were named more slowly and with higher error rates than the identical images. Given the sensitivity of LOC to simple object parts, retaining the intact object parts but in configurations that were uninterpretable as familiar visual entities would thus appear to be a relevant control for low-level information in assessing whether LOC was sensitive to familiarity.

## Methods

### Participants

Thirteen University of Southern California students (all right-handed, ten males, three females, mean age 20, range 19–24) participated in the experiment. All subjects were screened for safety and gave informed consent in accordance with the procedures approved by University of Southern California's Institutional Review Board.

### Stimuli

Seventy-two readily identifiable images of familiar objects were created using Blender (Blender Foundation, Blender Institute, Amsterdam, Netherlands). Each object was composed of three or more geons, with each geon corresponding to a simple part of the object. Novel versions of each object were constructed by using the same parts but rearranging them, i.e., altering the relations between the geons (Figure 1). By reconfiguring the relative geon positions in an object, the novel objects were thus constructed to be unrecognizable with respect to familiar object categories, while still controlling for stimulus properties known to be relevant to LOC. Properties that affect earlier stages of visual processing were also controlled by equating the pixel energy of each familiar-novel image pair, as described by Kayaert, Biederman, and Vogels (2003).

Scrambled versions of the objects, resembling texture, were constructed in MATLAB (The MathWorks, Natick, MA) by randomly permuting blocks of 3 × 3 pixels.

To assess the possible effect of symmetry, two raters judged the familiar and novel objects for object (not image) symmetry. Objects were classified as being symmetrical if there was a plane in 3D that could divide the object into mirror halves (see Figure 1). There was strong agreement between the raters—with a meeting between the raters in which discrepancies were readily resolved. Sixty-three of the familiar and twenty-six of the novel objects were judged to be symmetrical. Because of the greater number of symmetrical familiar objects, a post hoc analysis was conducted to evaluate the effects of symmetry.

### Procedure and fMRI design

Subjects participated in a total of two 216 s fMRI runs, each consisting of 18 blocks with three trial types—novel, familiar, and scrambled—presented in a pseudorandom block design. An entirely different set of stimuli was used for each run, allowing us to infer the encoding of familiarity in LOC using a correlational multivoxel pattern analysis. If there had been overlap in the stimuli used between runs, it would be unclear if effects revealed by the MVPA were due to familiarity, differences in shape, or both. The block history of two look-backs was balanced across all trial types, and the first block of each run was excluded from the statistical analysis to minimize nonsteady-state responses.

**Figure 2:**
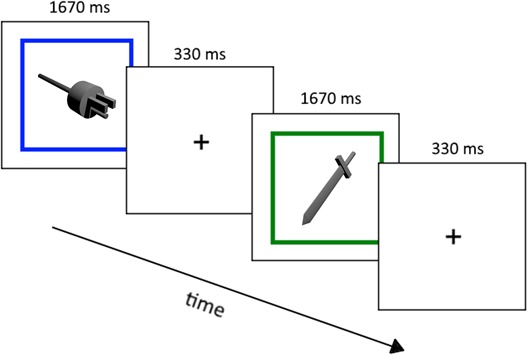
Illustration of the presentation sequence for two trials with familiar objects. For each trial, the fixation cue was displayed for 330 ms followed by a 1670 ms stimulus presentation with a total of six stimuli (trials) per block. The sequence above also demonstrates a border color change, which the subjects were asked to report via button press as an indicator of attentiveness.

In each block, six stimuli were presented by a video projector with a linear gamma. Presentation sequences of stimuli were programmed with Psychophysics Toolbox (Brainard, 1997; Pelli, 1997) running on MATLAB. Each stimulus was preceded by a 330 ms fixation cross, followed by the stimulus for 1670 ms in the center of the screen (Figure 2). Each object subtended a visual angle of approximately 3° × 3°. Subjects were instructed to passively view each object for the full duration it was displayed. During each run a colored border surrounded the stimuli. The color of the border was changed at a random time during the block. As an orthogonal task, subjects were to press a button when they noticed the color of the border change. Subjects were told that the main task was to view the stimuli and that the purpose of the border-color change detection task was to ensure that they were attentive while in the scanner.

Along with the two fMRI scans, a T1-weighted acquisition was performed for each subject to generate the anatomical overlay for within- and between-subjects coregistration.

### Data acquisition, imaging parameters, and data analysis

Scanning was performed at the Dana and David Dornsife Cognitive Neuroscience Imaging Center at the University of Southern California using a Siemens 3T MAGNETOM TIM Trio with a 12-channel coil. Responses were recorded with an MRI compatible button box.

High-resolution T1-weighted anatomical images and T2*-weighted functional images were acquired. The MPRAGE sequence was used for the anatomical scan with TR = 1950 ms, TI = 900 ms, TE = 2.26 ms, Flip angle = 9°, 160 sagittal slices, and 1 × 1 × 1 mm voxels. Echo planar imaging sequence was used for functional acquisition, with TR = 2000 ms, TE = 30 ms, FOV = 192 mm, flip angle = 90°, and voxel size 3 × 3 × 3 mm. 30 near-coronal slices, prescribed perpendicularly to the calcarine sulcus, were acquired in 12 of the 13 subjects. The remaining subject had an axial slice prescription.

The functional imaging data were analyzed using FSL (FMRIB's Software Library, www.fmrib.ox.ac.uk/fsl). 3D motion was corrected using MCFLIRT. Slice timing correction was performed using slice interpolation. A space-domain 3D spatial smoothing was performed using a 5 mm full-width at half-max Gaussian filter on all volumes. Each volume/sequence was filtered using a high pass filter set to 72 s (the length of six blocks).

FILM (FMRIB's Improved Linear Model) prewhitening was used to provide a robust and accurate nonparametric estimation of time series autocorrelation on each voxel's time series (Woolrich, Ripley, Brady, & Smith, 2001). FMRI data processing was carried out using FEAT (FMRI Expert Analysis Tool) Version 6.00, part of FSL. Registration to high resolution structural and MNI standard space images was carried out using FLIRT (Jenkinson, Bannister, Brady, & Smith, 2002).

### Voxel-wise analyses

For each run, activations were calculated in all acquired voxels using the general linear model. The two runs of each subject were combined via fixed effects analysis using FEAT. Mixed effects analysis across subjects utilized FEAT FLAME 1 & 2 (FMRIB's Local Analysis of Mixed Effects) to model and estimate the random-effects components of the measured intersession mixed-effects variance.

For visualization purposes, group-level findings in MNI space were projected onto a flattened representation of the cortex for each of the brain hemispheres using Freesurfer (http://surfer.nmr.mgh.harvard.edu/) and its average subject “fsaverage.”

### ROI analyses

Functional ROIs representing LOC were defined separately for each subject. Specifically, two bilateral ROIs were defined: the first based on the Familiar minus Scrambled contrast and the second based on the Novel minus Scrambled contrast. For both contrasts, clusters often extended into areas V2, V3, and V4, as defined by the PALS visuotopic annotations (Van Essen, 2005) in Freesurfer. Thus, to isolate the LOC ROI, clusters were rethresholded beyond the initial Z = 2.3 cutoff, such that clusters extending into areas V2, V3, and V4 could be distinct from clusters near areas LO and pFs. This process was conducted iteratively by inspection for each subject. The resulting maps contained a group of clusters that included areas LO and pFs but did not extend into other retinotopic areas, as well as a separate group of clusters that included retinotopic areas but did not include LO or pFs. Manual edits and parcellation enforcing this membership were made in volumetric space for individual subjects using fslview, and final results were inspected on the flattened cortex in Freesurfer. For each contrast, all clusters which included LOC but did not include the retinotopic areas were combined into a single ROI. The two ROIs (Familiar minus Scrambled and Novel minus Scrambled) were combined disjunctively to create a single LOC ROI for each subject, such that voxels in the final ROI were members of the Familiar minus Scrambled ROI, the Novel minus Scrambled ROI, or both. For ROI statistical analyses, mean activation values in terms of % BOLD signal change within the ROI for each contrast were compared.

### Multivoxel analysis

Given that each subject only had two functional runs, we had insufficient data to train a linear classifier on the patterns of voxel activity. Instead, to determine whether voxel activity patterns within the LOC functional ROIs differed between conditions of familiarity, we used an approach similar to that employed by Haxby et al. (2001) and computed within-conditions voxel correlations across the two runs (e.g., the correlation of multivoxel activity between viewing the Familiar stimuli in the first run and viewing Familiar stimuli in the second run) as well as between-conditions voxel correlations (e.g., the correlation of multivoxel activity between viewing of Familiar stimuli in the first run and viewing Novel stimuli in the second run). These correlations were calculated in MATLAB using contrasts of parameter estimates (Familiar-minus-Scrambled and Novel-minus-Scrambled) obtained from first level voxel-wise analyses in FEAT from individual subjects. The raw correlation values were down adjusted by computing the effective number of independent voxels based on the intrinsic smoothness of the voxel-wise contrast values, estimated using “smoothest” function in FSL. The difference in between- versus within-conditions correlations across subjects was assessed using a paired *t* test after transforming the *r* values to *z* scores using the Fisher transform.

## Results

### fMRI results

#### Novel versus familiar

**Figure 3:**
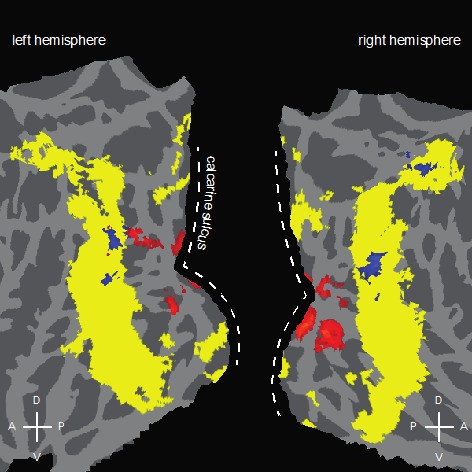
Group-level statistical map projected to a flattened representation of the cortices, showing [Intact (Familiar and Novel) > Scrambled], in yellow, which marks object-selective regions that include LOC. Red voxels are those with a significantly higher BOLD response for Novel than for Familiar. This novelty advantage was mostly confined to earlier visual areas with almost no activation in LOC. No voxel showed higher BOLD activation for Familiar compared to Novel intact objects. Blue voxels are those with a significantly higher BOLD response for Asymmetric (Familiar and Novel) than for Symmetric and are largely confined to the yellow regions and do not overlap with regions showing the novelty advantage.

As expected, relative to scrambled images, intact objects were associated with an elevated BOLD signal in a broad swath of lateral occipital regions bilaterally (shown in yellow in Figure 3), including LOC and bilateral regions of the parietal lobes (V3A, and superior parietal gyrus). We found no region showing elevated activity for familiar objects relative to novel ones. There were some regions, outside of those defined by Intact minus Scrambled (regions that included LOC), where novel objects produced more activity than familiar objects (shown in red in Figure 3). All of these were in earlier visual areas (V1–V3).

**Figure 4:**
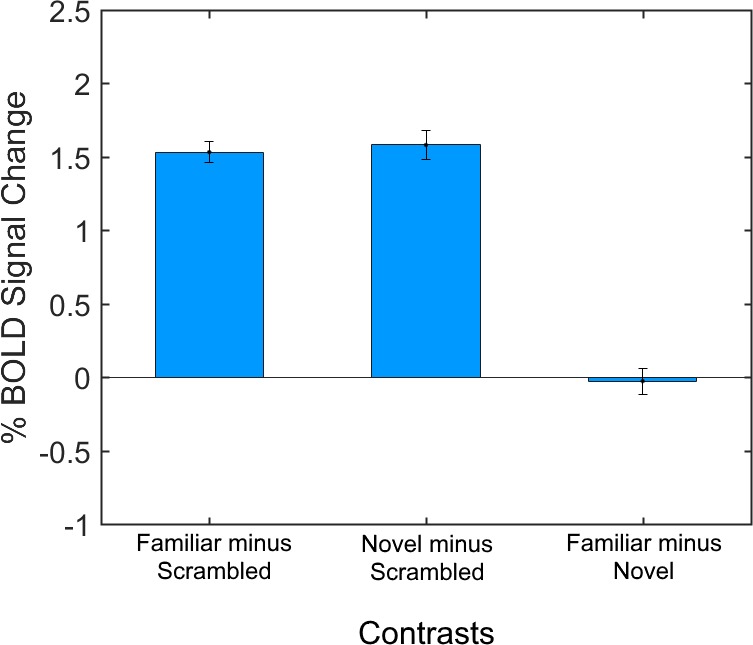
Mean percentage BOLD signal change across conditions in the LOC ROI. From left to right: Familiar minus Scrambled, Novel minus Scrambled, and Familiar minus Novel. Activation from both Familiar minus Scrambled and Novel minus Scrambled contrasts had mean percentage BOLD signal change significantly higher than zero, while activation from the Familiar minus Novel contrast did not.

Mean activity levels restricted to the functionally defined subject-wise LOC ROI were, as expected, significantly higher for the Familiar condition than for the Scrambled condition (mean BOLD signal change = 1.53% ± 0.07%, *p* < 10^−89^, Cohen's *d* = 6.1), and significantly higher for the Novel condition than for the Scrambled condition (mean BOLD signal change = 1.58% ± 0.10%, *p* < 10^−49^, Cohen's *d* = 4.4). There was no significant difference between mean activity levels for the Familiar and Novel conditions (mean BOLD signal change = 0.03% ± 0.09%, *p* = 0.77; Figure 4).

**Figure 5:**
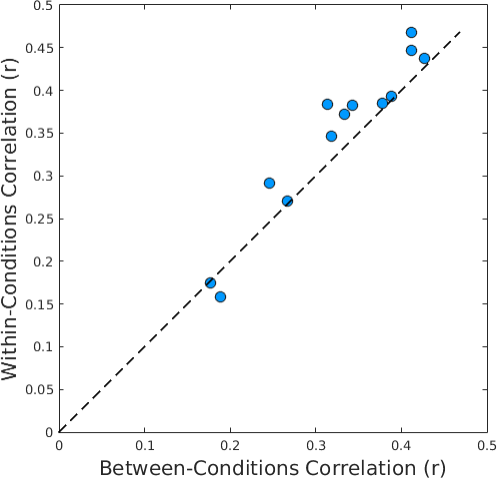
Comparison of within-conditions (Familiar vs. Familiar and Novel vs. Novel) smoothness-corrected correlations and between-conditions (Familiar vs. Novel) smoothness-corrected correlations for each subject. Eleven of the 13 subjects were above the identity line, showing higher voxel pattern correlations within conditions than between conditions.

A multivoxel correlational analysis revealed that the correlation within conditions (i.e., Familiar vs. Familiar and Novel vs. Novel, mean smoothness-corrected *r* = 0.347, mean uncorrected *r* = 0.703) was significantly higher than the correlation between conditions (i.e., Familiar vs. Novel, mean smoothness-corrected *r* = 0.324, mean uncorrected *r* = 0.674) within-subjects paired *t* test on the Fisher Z-transformed smoothness-corrected *r* values: *t*(12) = 3.3, *p* = 0.0063, Cohen's *d* = 0.79. Specifically, as shown in Figure 5, 11 of the 13 subjects had higher within-conditions correlations than their between-conditions correlations.

#### Symmetry versus asymmetry

Because there were a greater number of asymmetrical objects in the novel versus the familiar condition, 63 versus 26, respectively, we performed a voxel-wise analysis to assess whether the symmetry of the stimuli had any differential effect on the magnitude of the BOLD response. Greater activation was found for asymmetrical objects, shown in blue in Figure 3, in a few scattered regions in LOC and the parietal regions that had greater activation to intact objects. (Because the sequences were randomized, rather than balanced, with respect to symmetry this inference must be made tentatively.) These asymmetry-preferred regions did not overlap with regions preferring novelty, shown in red in Figure 3, which were outside of LOC. Indeed, when the voxelwise familiar versus novel comparison was performed with either asymmetrical objects only (i.e., Familiar Asymmetrical minus Novel Asymmetrical) or symmetrical objects only (i.e., Familiar Symmetrical minus Novel Symmetrical), no voxel clusters in LOC or in early visual areas showed reliable increased activity for either condition of familiarity.

#### Orthogonal task response times

Mean RTs for the orthogonal task of detecting a color change in the border was 633 ms. On average, each subject missed one border color change over the two runs. The difference in RTs to the border color change task during the different block types was not significant, repeated-measured ANOVA *F*(2, 24) = < 1.00. These results suggest that the subjects were attentive to the stimuli during the experiment and their attention was not affected by stimulus type. Attentional engagement would be expected given that recognition of objects is automatic Smith & Magee, 1980). That is, it is impossible to look at, say, a bicycle and not know what it is.

## Discussion

Our results confirm Malach et al.'s (1995) contention that there is no net mean-BOLD effect of object familiarity on overall LOC activation. Certainly there was no evidence that familiar objects produced greater activity in LOC than their novel controls. Intact objects, whether novel or familiar, produce greater activation in LOC than scrambling, also as reported by Malach et al. as well as hundreds of subsequent studies. We achieved much stricter control over familiar versus novel stimuli by having the novel objects generated by a simple rearrangement of the parts of the familiar objects. This procedure also allowed the contrast energy to be equated between novel and familiar images. Since our functional acquisitions did not cover more anterior regions of the ventral pathway, such as the anterior portions of the temporal lobes or PFC, we were unable to observe whether these anterior regions would show greater activity to familiar objects.

Because our objects were composed of well-defined simple parts (geons) with a uniform surface rendering, they lacked both the irregularities typical of many real world objects as well as their characteristic material and surface properties. Could this lack of verisimilitude be responsible for the absence of a net effect of familiarity? That is, were subjects actually able to identify the objects and judge them as familiar versus novel? To address this question, we had 15 subjects, who had not participated in the main experiment, view the stimuli and judge whether they were familiar or not. The response (a key press) terminated the stimulus presentation. The mean accuracy in judging the images was 91.5% ± 2.47%, significantly greater than chance. A one-sample *t* test relative to the chance level of 50%, *t*(14) = 23.7, *p* < 10^−19^, Cohen's *d* = 1.40, suggests that the subjects could reliably distinguish familiar from novel. These judgments were made in a mean response time of 1416 ± 242 ms, which was less than the 1670 ms stimulus duration in the fMRI task. Thirteen of the 15 subjects had mean response times less than 1670 ms.

There was somewhat greater activation outside of LOC in early visual areas for Novel as compared to Familiar objects. We speculate that novel objects invite inspection as an observer might attempt to derive the possible functionality of such objects or, alternatively, try to infer what familiar object served as the origin of the image (although the subjects were not informed as to how the novel objects were generated).

The MVPA results suggest that there is familiarity coding in LOC. A recent multivoxel pattern analysis by Iordan, Greene, Beck, & Fei-Fei (2015) found that a classifier based on LOC voxels correctly classified the basic, subordinate, and superordinate category membership of 32 stimuli, composed of eight dogs, flowers, airplanes, and shoes. This result, in conjunction with the present findings, suggests that whereas the pattern of voxel activity in LOC may vary with familiarity, the different patterns sum to approximately equivalent overall magnitudes regardless of familiarity.

Somewhat inconsistent with Iordan's MVPA results, however, are two fast event-related fMRI studies (Chouinard, Morrissey, Kohler, & Goodale, 2008; Kim, Biederman, Lescroart, & Hayworth, 2009) in which subjects viewed a sequentially presented pair of objects. A repetition of the identical image resulted in adaptation in that the BOLD response was diminished compared to when a different image was shown. However, when the different image was of a different basic-level category, e.g., dog1 followed by monkey1, the release from adaptation was no greater than when the two images were of the same basic-level category, e.g., dog1 followed by dog2. This held true even though the physical dissimilarity of dog1 and dog2 was equal to the dissimilarity of dog1 and monkey1, as scaled by the Gabor-jet model (Lades et al., 1993; Yue, Biederman, Mangini, von der Malsburg, & Amir, 2012), a model that captures the multiscale, multiorientation tuning of V1 simple cells. A possible resolution to this inconsistency could rest on the assumption that if LOC had different pattern activities at the exemplar (subordinate) levels, then the trained classifier could simply regroup these distinctive patterns. If this view is correct, then the Iordan et al. result does not speak to semantic coding in LOC. Future research will be required to assess this apparent discrepancy between MVPA and Event-Related Adaptation paradigms. All in all, the current results support the notion that LOC is the end stage in the ventral pathway where a physical representation of an object is achieved. By this account, the access to semantics that could affect performance occurs at a later stage.

## Supplementary Material


