# Supplementary figures and images for: The Lateral Occipital Complex shows no net response to object familiarity
Source: J Vis. 2016 Sep 6;16(11):3. doi: 10.1167/16.11.3 (PMC5024672; doi:10.1167/16.11.3)

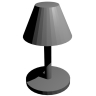

Supplement: Supplementary file 1 [file i1534-7362-16-11-3-icon01.gif]
